# *In Utero* Exposure to Benzo[*a*]Pyrene Increases Mutation Burden in the Soma and Sperm of Adult Mice

**DOI:** 10.1289/EHP211

**Published:** 2016-07-22

**Authors:** Matthew J. Meier, Jason M. O’Brien, Marc A. Beal, Beverly Allan, Carole L. Yauk, Francesco Marchetti

**Affiliations:** 1 Environmental Health Science and Research Bureau, Health Canada, Ottawa, Ontario, Canada; 2 Department of Biology, Carleton University, Ottawa, Ontario, Canada

## Abstract

**Background:**

Mosaicism, the presence of genetically distinct cell populations within an organism, has emerged as an important contributor to disease. Mutational events occurring during embryonic development can cause mosaicism in any tissue, but the influence of environmental factors on levels of mosaicism is unclear.

**Objectives:**

We investigated whether in utero exposure to the widespread environmental mutagen benzo[a]pyrene (BaP) has an impact on the burden and distribution of mutations in adult mice.

**Methods:**

We used the Muta™Mouse transgenic rodent model to quantify and characterize mutations in the offspring of pregnant mice exposed to BaP during postconception days 7 through 16, covering the major period of organogenesis in mice. Next-generation DNA sequencing was then used to determine the spectrum of mutations induced in adult mice that were exposed to BaP during fetal development.

**Results:**

Mutation frequency was significantly increased in the bone marrow, liver, brain, and sperm of first filial generation (F1) males. Developing embryos accumulated more mutations and exhibited higher proportions of mosaicism than exposed adults, particularly in the brain. Decreased sperm count and motility revealed additional negative impacts on the reproductive function of F1 males.

**Conclusion:**

In utero exposure to environmental mutagens contributes to somatic and germline mosaicism, permanently affecting both the genetic health of the F1 and the population gene pool.

**Citation:**

Meier MJ, O’Brien JM, Beal MA, Allan B, Yauk CL, Marchetti F. 2017. In utero exposure to benzo[a]pyrene increases mutation burden in the soma and sperm of adult mice. Environ Health Perspect 125:82–88; http://dx.doi.org/10.1289/EHP211

## Introduction

Our understanding of human genetic disease is predicated on the idea that most mutations are inherited through the germline. However, mounting evidence suggests that disease-associated genetic changes also arise during embryonic development (Biesecker and Spinner 2013; Erickson 2010; Lupski 2013, 2015). These postzygotic events (which may include mutations, large-scale rearrangements, or aneuploidies) produce a varied distribution of altered genomes throughout the individual—a phenomenon known as mosaicism (De 2011). Any cell type in the body can accumulate such mutations, including stem cells or primordial germ cell precursors, which induce permanent changes in individuals or in their offspring (Campbell et al. 2015; Cohen et al. 2014; Rahbari et al. 2016). Recent genome-scale studies have revealed unexpected levels of mosaicism in seemingly normal tissues (Campbell et al. 2015; Erickson 2010; Rahbari et al. 2016), and we may still be vastly underestimating the prevalence and health burden of low-level mosaicism (Campbell et al. 2014; Spinner and Conlin 2014).

Recent work by Rahbari et al. (2016) exemplifies the prevalence of mutations occurring during early development and their significant contribution to somatic and germline mosaicism in adults. The authors also provide compelling evidence that germline mutation rates are not constant throughout the lifetime of an organism, and that spontaneous mutation may be more likely to occur during the expansion of male primordial germ cell precursors in embryogenesis than during other developmental stages or post-pubertal spermatogenesis. Although individual differences were observed in the prevalence of germline mosaicism, no studies since the pioneering work of Russell et al. (Russell and Russell 1992; Russell 1999; Russell et al. 1988) have investigated whether exposure to environmental factors during development alters the induction of mosaic mutations in the germline. This previous work demonstrated, using phenotypic markers in the specific locus test, that the perigametic interval was a significant source of spontaneous as well as chemical- or radiation-induced mosaic mutations. However, other stages of development, such as fetal growth, have remained uncharacterized with respect to the induction of mosaicism by environmental factors. In general, mutation assays measure both unique and clonally expanded mutations; however, the relative contribution of mosaicism to the overall mutation burden is rarely considered in these assays. Moreover, the influence of mutagen exposure during critical developmental stages on the degree of tissue-wide mosaicism remains a significant gap in understanding in the field of genetic toxicology.

*In utero* exposure to toxicants can cause a range of deleterious health effects in adulthood [e.g., reproductive defects (Fowler et al. 2008; Mocarelli et al. 2011), increased cancer susceptibility (Autrup 1993), impaired cardiac function (Buscariollo et al. 2014), and neurodegenerative disease (Modgil et al. 2014)]. However, the extent to which *in utero* exposure to environmental chemicals contributes to adult disorders resulting from the induction and distribution of mutations in developing tissues is unknown (Ritz et al. 2011). We used a transgenic rodent model combined with next-generation sequencing to investigate the effects of *in utero* exposure to benzo[*a*]pyrene (BaP), a common environmental pollutant and human carcinogen produced by a variety of sources, on the burden and distribution of mutations in adults. This study presents the first evidence that fetal exposure of mice to a mutagenic chemical can directly result in an excess burden of mutations and increased mosaicism in both somatic tissues and germ cells of adult first filial generation (F1) mice.

## Methods

Mutagenicity data, statistical analysis, and detailed methodology described in this paper are available in the Supplemental Material. Sequence data for *lacZ* mutants are archived in the NCBI sequence read archive under BioProject PRJNA342797.

### Animal Treatment

The use of animals in these experiments was approved by the Health Canada Ottawa Animal Care Committee. Animals used in this study were humanely treated with regard to the alleviation of suffering following the guidelines of the Canadian Council on Animal Care (http://www.ccac.ca/en_/standards/policies/policy-ethics_animal_investigation). Male and female Muta™Mouse mice were obtained from a colony maintained at Health Canada. Males were housed with up to four females, and every morning females were checked for the presence of a vaginal plug as indication of mating. Pregnant mice were dosed with 0, 10, 20, or 40 mg/kg/day BaP (Sigma-Aldrich Canada Ltd) dissolved in olive oil (at a volume of 0.15 mL for 30 g body weight) and administered on postconception days 7 through 16 by oral gavage (with postconception day 1 indicated by the presence of a vaginal plug). Each pregnant female was housed individually. Pups were weaned at 3 weeks of age and euthanized 10 weeks after birth in accordance with Health Canada’s ethical guidelines, after which tissues were collected. The bone marrow, brain, liver, testis, and cauda epididymis (one per mouse) were flash frozen in liquid nitrogen immediately following necropsy and were stored at –80°C until DNA extraction was performed.

### *lacZ* Transgene Mutation Assay

The *lacZ* transgenic rodent mutation assay was performed as previously described, in a manner consistent with Organisation for Economic Co-operation and Development (OECD) Test Guideline 488 (O’Brien et al. 2014; OECD 2011). DNA was isolated from tissues by phenol/chloroform extraction and packaged into lambda phage (Transpack packaging extract, Agilent Technologies). The packaged reporter constructs were subsequently plated on a lawn of *Escherichia coli* (*galE* ^–^) grown on lysogeny broth (LB) media, and positive selection for mutant plaques was performed using phenylgalactoside (Gossen et al. 1992). Mutant plaques were counted, and a subset of plaques were collected in MilliQ (EMD Millipore Corporation) water (3 μL per plaque) for sequencing. The dose–response of mutant frequency was tested for significance with generalized linear modeling in R using a quasi-Poisson distribution (version 3.2.1; R Project for Statistical Computing) as well as dose–response modeling using both the R package PROAST (http://www.rivm.nl/en/Documents_and_publications/Scientific/Models/PROAST) and benchmark dose software [BMDS v.2.6 from the U.S. Environmental Protection Agency (EPA); http://www.epa.gov/ncea/bmds/].

### Computer-Assisted Sperm Analysis

Computer-assisted sperm analysis (CASA) was performed on an IVOS instrument (Hamilton Thorne, Inc.) using sperm from one of the cauda epididymides taken from mice at the time of necropsy. Each cauda was minced into 2.5 mL of prewarmed M16 or M199 medium (Sigma) for 10 mg/kg/day BaP or for 20 and 40 mg/kg/day BaP, respectively. After 3 min at 37°C in 5% CO_2_, 16 μL of a 1:4 dilution of sperm was added into the two wells of a 2X-CEL 80-μm-deep chambered slide (Hamilton Thorne, Inc.). At least 10 fields per chamber, automatically selected by the IVOS instrument, were imaged with a 4× objective and analyzed with IVOS Animal software v.14 (Hamilton Thorne, Inc.). The settings for CASA analysis were: frame rate, 60Hz; 30 frames acquired/samples; minimum contrast, 40; minimum cell size, 3.

### Statistical Analyses

The *p*-values for body weight, liver somatic index (LSI), testis somatic index (TSI), and sperm motility metrics were determined using an analysis of variance (ANOVA) followed by a Bonferroni post hoc multiple comparison relative to the control group. Dose–response data from the *lacZ* transgene assay were analyzed in R using the glm function. The number of mutant plaques was compared to dose, setting log(total plaques) as the offset and using the quasi-Poisson distribution family to account for over-dispersion. The resulting *p*-values were corrected for multiple comparisons using the Bonferroni method. A likelihood ratio test was used to eliminate outliers between technical replicates (within animals), and subsequently between animals within dose groups. Dose–response analysis for all tissues was performed using all available models in BMDS v.2.6 (http://www.epa.gov/ncea/bmds/), followed by selection of the best-fit model using the Akaike information criterion (AIC).

### Ion Proton Sequencing

Pooled plaques collected from the transgenic rodent assay were subjected to polymerase chain reaction (PCR) after heating at 95°C and centrifugation to remove *E. coli* cellular debris. PCR was performed in duplicate technical replicates for each sample using NEB Phusion DNA Polymerase (New England Biolabs) according to the manufacturer’s instructions. PCR-amplified DNA was purified with a QIAquick PCR purification kit (QIAGEN) and then used to create a fragmented DNA library via ligation to P1 adapter and barcoded A adapter using the NEBNext® Ion Kit (New England Biolabs). The resulting libraries were pooled in equimolar quantities after quantification on an Agilent Tapestation D1000 (Agilent Technologies). These libraries were used in template preparation on an Ion Chef™ robot (Thermo Fisher Scientific Inc.) and sequenced using a P1 chip on an Ion Proton™ (Thermo Fisher Scientific Inc.) instrument.

### Computational Analyses

Mutations were called as previously described (Beal et al. 2015). Reads were aligned to the *lacZ* sequence from Muta™Mouse using bowtie2 with the “very sensitive local” option enabled. Pileups were created using samtools mpileup v.0.1.19 (http://samtools.sourceforge.net/mpileup.shtml), and mutations were called with a customized R script (available online at http://usegalaxy.org). Based on the pileups, a proportion of each base call at each position of the *lacZ* gene was determined for each library (each of which constitutes a different sample of pooled plaques, performed in technical replicate). Putative mutations were filtered based on the following criteria: they must be present above the pooled mutation calling threshold (1/number of plaques sequenced) in both technical replicate DNA libraries, and the background rate of the mutation must be < 2%. For calculating clonal expansion, the count of each mutant was determined by multiplying the percentage of reads containing each unique mutation by the number of plaques sequenced for that animal; because we observed high variability among low numbers of mutant counts, we applied a limit of detection/linear model to correct the counts (Beal et al. 2015). This method relies on the conservative assumption that any two reads derived from the same biological sample and possessing an identical mutation within one tissue are likely to be the product of clonal expansion rather than independent events. Clonally expanded mutants were then considered to be those with a corrected count > 1, and mutants with a corrected count equal to 1 were considered singletons. Mutation spectra were generated using the counts for each unique mutation type.

## Results

### Mutations in Somatic Tissues after *in Utero* BaP Exposure

To determine the genetic effects of transplacental exposure to an environmental mutagen, BaP was administered in olive oil at 0, 10, 20, or 40 mg/kg/day by oral gavage to pregnant Muta™Mouse females on postconception days 7–16, comprising the period of organogenesis in mice (Mitiku and Baker 2007). These doses were chosen based on previous reports in the literature indicating that such an exposure affected the fertility of the F1 generation (MacKenzie and Angevine 1981). Neither litter size at birth nor body weight of the F1 generation (at 10 weeks of age) was significantly affected by BaP administered during pregnancy, indicating that BaP exposure in the dam did not cause embryo loss or have a significant impact on postnatal development.

**Figure 1:**
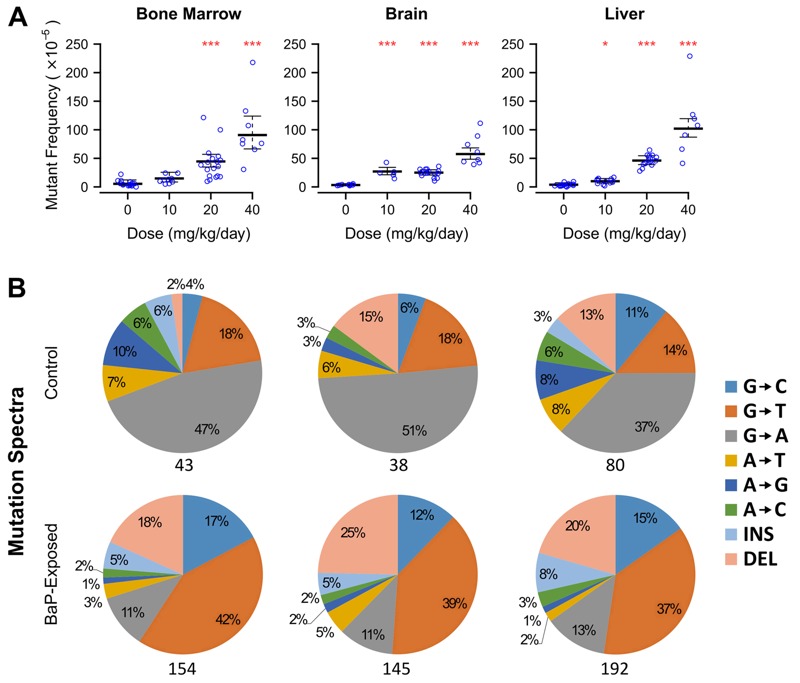
Male mice exposed transplacentally to benzo[*a*]pyrene (BaP) during organogenesis exhibited increased mutant frequencies in multiple somatic tissues. Dams were dosed at 0, 10, 20, or 40 mg/kg/day, and mutant frequencies [in 10-week-old first filial generation (F1) males] were determined using the *lacZ* transgene mutation assay. (*A*) *In utero* exposure to BaP induced a significant dose-dependent increase in mutant frequencies in somatic tissues originating from distinct primary germ layers, including the bone marrow (mesoderm), brain (ectoderm), and liver (endoderm). Error bars represent 95% confidence intervals. *p < 0.05, ***p < 0.0001, generalized linear model with quasi-Poisson distribution. (*B*) Next-generation sequencing of the *lacZ* reporter gene from animals exposed *in utero* shows the well-characterized molecular signature of BaP exposure, indicated in the pie charts as an increase in the proportion of G→T mutations. The number of unique *lacZ* mutations sequenced is noted beneath each pie chart. INS, insertion; DEL, deletion.

We then measured mutations in three somatic tissues derived from each germ layer (ectoderm: brain; mesoderm: bone marrow; endoderm: liver) using the recoverable *lacZ* reporter transgene within the Muta™Mouse genome (Lambert et al. 2005). Transplacental BaP exposure induced dose-dependent increases in mutations in the somatic tissues of F1 males (Figure 1; see also Table S1). At the highest dose, mutant frequencies increased 16-, 18-, and 33-fold (*p* < 0.0001) above controls in bone marrow, brain, and liver, respectively. At the low dose, brain showed a 7-fold increase in mutant frequency (*p* < 0.0001), liver showed a 3-fold increase (*p* = 0.04), and bone marrow was unaffected (*p* = 0.13). Although BaP adduct levels are typically elevated in maternal tissues relative to fetal tissues (Lu et al. 1986), *in utero* exposed liver and brain had 3- and 19-fold higher numbers of mutants per milligram/kilogram body weight than adult BaP-exposed tissues, respectively (see Figure S1 and Table S2). Conversely, the mutagenic response in bone marrow did not differ between *in utero* and adult exposures. This outcome is likely because all tissues are mitotically active during *in utero* development and therefore are susceptible to mutation fixation; in contrast, adult liver and brain have very low mitotic indices. These results indicate that the placenta does not shelter the embryo from polycyclic aromatic hydrocarbons and that embryonic development is a highly susceptible window for the induction of somatic mosaicism.

### Effects of *in Utero* BaP Exposure on Reproductive Health

**Figure 2:**
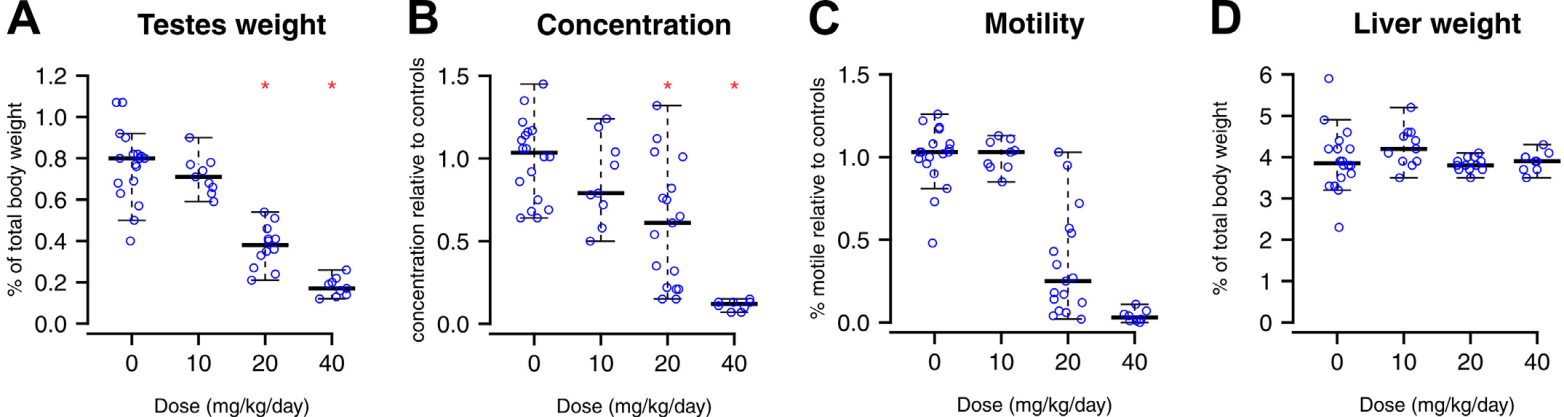
*In utero* exposure to benzo[*a*]pyrene (BaP) significantly decreases multiple reproductive parameters in first filial generation (F1) adult male mice. BaP caused a significant dose-dependent decrease in the testis weight (shown as percentage body weight) (*A*), as well as a drastic decrease in sperm concentration (*B*) and percentage of motile sperm (*C*). Liver weight (*D*) was unaffected. *p < 0.05, analysis of variance (ANOVA). Error bars represent 95% confidence intervals.

**Figure 3:**
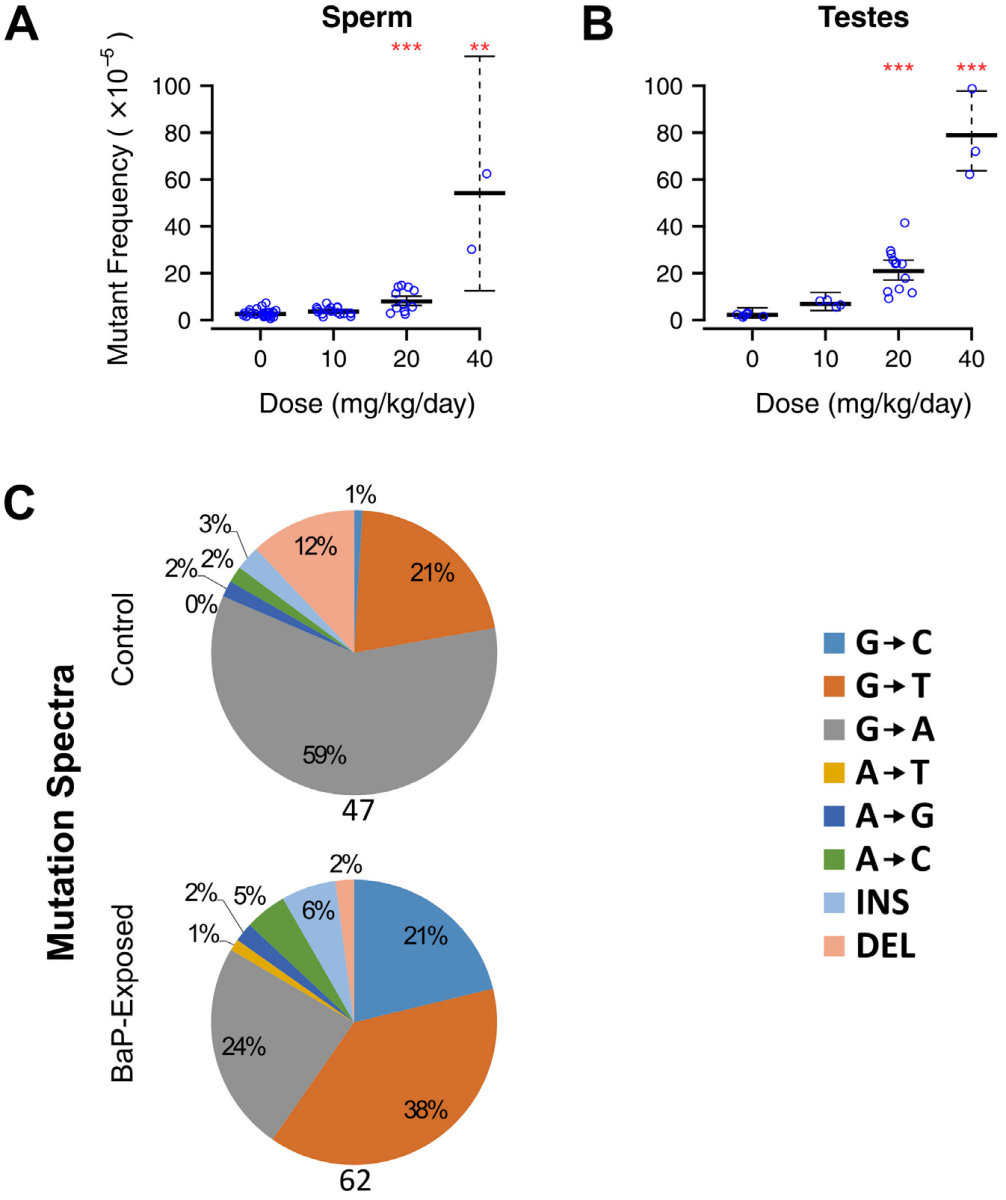
Mutant frequencies were significantly increased in caudal sperm (*A*) and whole testes (*B*) of first filial generation (F1) males exposed *in utero* to 20 and 40 mg/kg/day benzo[*a*]pyrene (BaP). Mutant frequencies in sperm at 40 mg/kg/day were measured by pooling several DNA samples because of extremely low sperm counts. Error bars represent 95% confidence intervals. **p < 0.001, ***p < 0.0001, generalized linear model with quasi-Poisson distribution. (*C*) Next-generation sequencing of the *lacZ* reporter gene from caudal sperm. BaP exposure (20 and 40 mg/kg/day combined) increased the proportion of G→T and G→C mutations but had no effect on deletions. The number of unique *lacZ* mutations sequenced is noted beneath each pie chart.

Next, we assessed the effects of *in utero* exposure to BaP on the reproductive health of the offspring. Although no effects occurred at 10 mg/kg/day, we observed dose-related effects on all male reproductive parameters at the other doses. At 40 mg/kg/day, testis weight decreased by 77% (Figure 2A), and sperm concentration decreased > 90% (Figure 2B). Computer-assisted sperm analysis indicated that *in utero* BaP exposure significantly decreased all of the measured motility parameters (Table 1, Figure 2C). Furthermore, *in utero* exposure to 20 mg/kg/day BaP caused a significant increase (3-fold, *p* < 0.0001) in *lacZ* mutants in sperm (Figure 3A). At 40 mg/kg/day, so few sperm were present in the cauda epididymis that samples required pooling to obtain sufficient DNA for mutation analysis. These pooled samples had significantly higher mutant frequencies than lower doses and controls (Figure 3A; 16-fold, *p* = 0.0003), and the average mutant frequency (46.3 × 10^–5^) was 4-fold higher than that seen in sperm of adult males exposed to 100 mg/kg/day *N*-ethyl-*N*-nitrosourea (ENU) (O’Brien et al. 2015), the most potent germ cell mutagen known. This equates to 11-fold higher mutants per milligram/kilogram body weight of BaP in sperm from mice exposed *in utero* than in sperm from exposed adults (O’Brien et al. 2016a). Mutant frequencies in whole testes (Figure 3B) closely matched those in sperm.

**Table 1:** *In utero* exposure to BaP negatively impacts sperm quality in F1 males as measured by computer-assisted sperm analysis.

Parameter	Fraction of control values^*a,b*^		
	10 mg/kg/day	20 mg/kg/day	40 mg/kg/day
Progressive motility	1.02	**0.36**	**0.02**
Path velocity (VAP)	1.03	0.77	**0.19**
Progressive velocity (VSL)	1.04	0.85	**0.20**
Track velocity (VCL)	1.01	0.70	**0.18**
Amplitude lateral head (ALH)	1.03	**0.66**	**0.16**
Beat cross frequency (BCF)	0.98	0.90	**0.50**
Straightness (STR)	1.00	1.07	**0.64**
Linearity (LIN)	1.01	1.21	0.76
^***a***^Bold indicates statistical significance of *p* < 0.05 in analysis of variance (ANOVA) followed by a Bonferroni post hoc multiple comparison. ^***b***^Nonnormalized data are available in Table S4.			

### Clonal Expansion of Mutations and the Induction of Mosaicism

**Figure 4:**
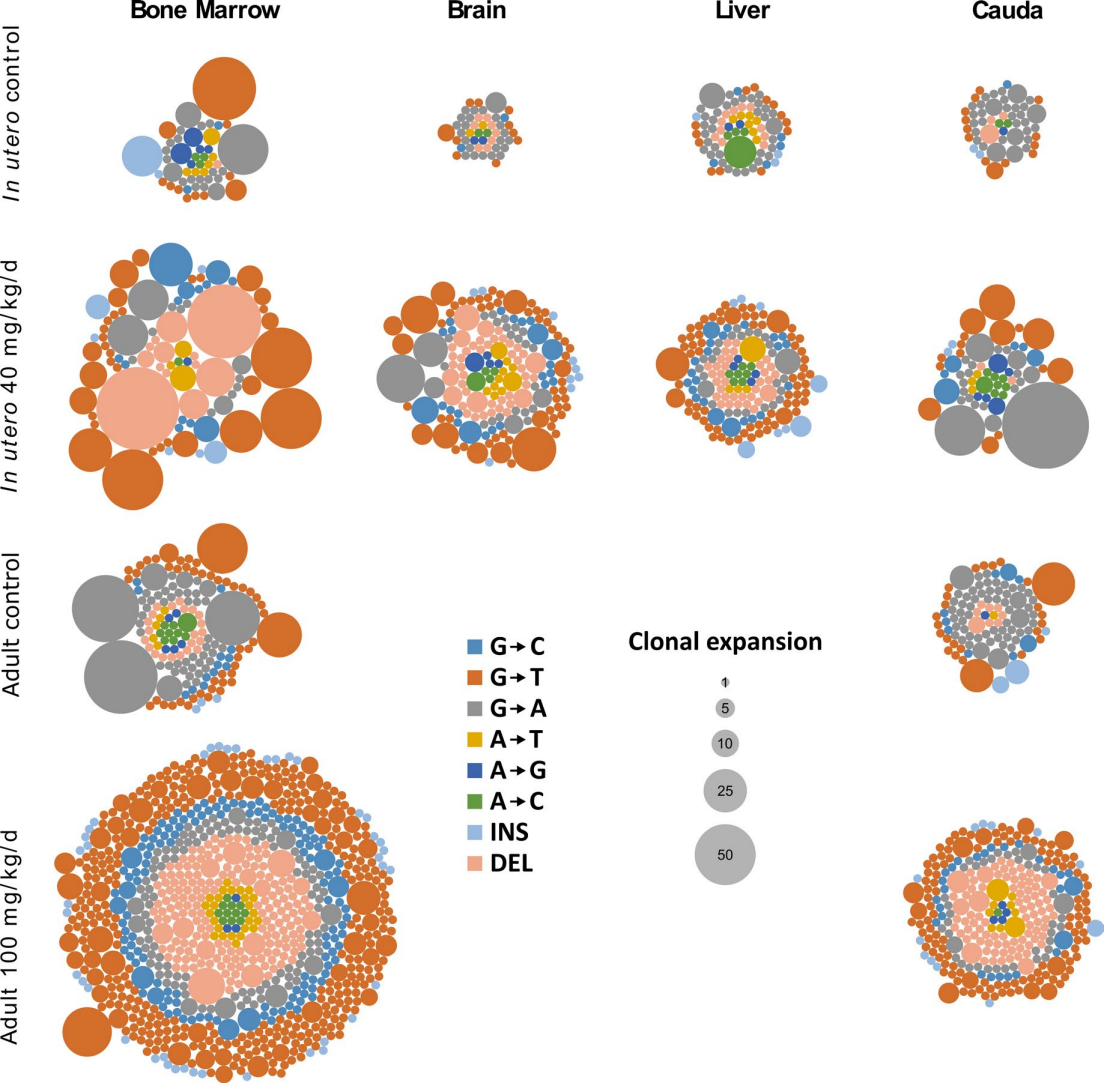
Clonal expansion of the mutations characterized in each somatic tissue and in caudal sperm. The proportion of mutations that underwent clonal expansion is consistently higher in animals exposed to benzo[*a*]pyrene (BaP) *in utero*, illustrating the potential for increased mosaicism. As in somatic tissues, the proportion of clonally expanded mutations is higher in BaP-exposed sperm, highlighting the potential for increased germline mosaicism. Compared with exposure in adults (data from Beal et al. 2015 and O’Brien et al. 2016b), *in utero* exposure results in a higher degree of clonal expansion. Plaques were collected from the 20 mg/kg/day dose group for cadual sperm because of decreased sperm concentration at 40 mg/kg/day. Each circle within a group corresponds to a scored independent mutation, and the area of each circle represents the number of times that mutation was observed per animal. INS, insertion; DEL, deletion.

We used next-generation sequencing to determine the mutation spectra induced by transplacental BaP exposure and to assess clonal expansion in both somatic tissues and sperm. We characterized 233 and 648 unique *lacZ* mutations from controls and BaP-treated tissues, respectively. In all four tissues, BaP exposure increased the proportions of *lacZ* mutations that were clonally expanded (Figure 4; see also Table S3). Interestingly, clonality correlates with the adult replication rate of each tissue, with bone marrow and sperm (as a proxy for spermatogonial stem cells) showing the highest clonality and having higher adult proliferation rates [19% and 35% dividing cells, respectively (Edwards and Klein 1961)]. Conversely, brain and liver showed the lowest clonality, with adult proliferation rates of neurons being negligible [< 0.1% dividing (Edwards and Klein 1961)] and hepatic cells dividing very rarely [0.53% dividing (Edwards and Klein 1961)]. These findings suggest that mutations were induced in stem cells during embryonic development and that the chance of detecting cells carrying the same mutation is affected by the rate of stem cell division in that tissue during adulthood.

Analysis of the mutation spectra provided insights into the mechanism(s) of BaP mutagenesis *in utero* and whether these differ among tissues. Because BaP mainly induces G→T transversions via mispairing of adducted DNA (Keohavong and Thilly 1992), increased proportions of G→T (as well as G→C transversions and deletions) were observed (Figure 1B). Mutation types were consistent among the three somatic tissues and with the mutation spectrum observed in the bone marrow of adult-exposed mice (Beal et al. 2015). The sperm spectrum was similar, except deletions were not increased (Figure 3C). Thus, the reactive metabolite of BaP (benzo[*a*]pyrene-7,8-dihydrodiol-9,10-epoxide) was likely active through a similar genotoxic mechanism in fetal tissues as in adults. The presence of the mutational signature of BaP also confirms that the increased proportion of *lacZ* mutations is caused by the reactivity of the chemical itself and not through induction of genomic instability.

## Discussion

We have demonstrated that BaP exposure during fetal development causes severe somatic and germ cell consequences in F1 animals and that tissues originating from all three germ layers, as well as the germ cells themselves, are highly mutagenized in adult offspring. Thus, *in utero* development is a critical window of risk for environmentally induced mutations that can lead to adverse health effects during adult life.

Historically, the discussion of somatic mutations has revolved around their role in tumorigenesis. In light of evidence that the number of lifetime stem cell divisions is a major contributor to cancer formation (Tomasetti and Vogelstein 2015), any first-hit mutations in critical genes taking place *in utero* would increase the likelihood for loss of heterozygosity in the affected locus. Because only three to six mutated driver genes are sufficient to initiate most cancers (Vogelstein et al. 2013), increased numbers of *de novo* mutations could accelerate cancer progression later in life. Furthermore, extrinsic risk factors such as environmental exposures may contribute 70–90% of lifetime cancer risk, suggesting—in the context of data presented in this study—that variation in developmental exposure to environmental mutagens plays a meaningful role in determining an individual’s risk (Wu et al. 2016). However, because mutations arising during *in utero* development are often observed as widely distributed mosaic variants, they can also result in diseases other than cancer.

Mosaicism in the nervous system is associated with diseases including epilepsy, lissencephaly, hemimegalencephaly, early-onset Alzheimer disease, autism spectrum disorders, and others (Poduri et al. 2013; Sanders et al. 2012; Yuen et al. 2015). Normal brains also possess distinct cell lineages with genetic variants that originate during development (Lodato et al. 2015). Here, the brains of mice exposed *in utero* showed a higher mutagenic response at lower doses of BaP than other tissues (*p* < 0.01), providing a novel mode of action for neurological effects induced by chemical exposures. We estimate that BaP-induced mutations increase by one standard deviation in the brain at 0.68 mg/kg-bw for a 10-day exposure (see Figure S2). This is a relatively small cumulative dose of 6.8 mg/kg-bw. In comparison, cancer induction in mice is estimated to occur at 0.8 mg/kg-bw over 2 years (a cumulative dose of 584 mg/kg-bw) (Moffat et al. 2015). Therefore, mutations in the developing brain are induced by lower cumulative doses of BaP than those required for adult cancer formation. Coupled with the observation that *in utero* BaP exposures as low as 0.3 mg/kg/day impaired neuronal activity in rats (McCallister et al. 2008), the developing brain demonstrates unprecedented sensitivity to BaP (Chepelev et al. 2015).

Developing germ cells differed in their response from developing somatic tissues in two major aspects: increased sensitivity to cell killing and mutation spectrum. The reduced sperm count in exposed offspring demonstrates increased sensitivity to BaP-induced apoptosis of primordial germ cell precursors (PGCs) via cellular toxicity, consistent with the framework proposed by Rübe et al. (2011). This evidence is supported by the results of a study in *Caenorhabditis elegans* that found somatic cells to be more resistant to DNA damage–induced apoptosis than germ cells (Gartner et al. 2000). Because PGCs were proliferating during the exposure window in the present study, PGC toxicity likely reduced the pool of germline stem cells in the offspring, and, consequently, caused a drastic and permanent reduction in sperm count. Furthermore, the BaP-induced mutation spectrum in sperm was significantly different from other tissues (Fisher’s exact test, Bonferroni adjusted, *p* < 0.001 compared with liver and brain; *p* = 0.097 compared with bone marrow), showing fewer deletions. Germline stem cells are thought to be more effective at repairing DNA damage because the integrity of their genome is vital to the faithful replication of DNA for the organism’s descendants (Rübe et al. 2011). It is therefore possible that deletions in sperm were under high selective pressure. These findings suggest that developing germ cells are more susceptible to environmental chemicals than adult spermatogonial stem cells, which may partially explain the higher mutation rate in primordial germ cells observed by Rahbari et al. (2016), and that the health effects of *in utero* mutagen exposure affect not only the exposed individual, but future generations as well.

Recent research has suggested that a large proportion of apparent *de novo* mutations in humans are in fact the result of low-level somatic and/or germline mosaicism in the parents (Rahbari et al. 2016). In a similar manner, our study used clonal expansion of independent transgene mutations as a method to quantify mosaicism. Figure 4 shows the relative quantity of each recovered mutation, where the size of the bubble correlates with the number of cells carrying that mutation in a given tissue. The transgenic rodent *in vivo* mutation assay positively selects *lacZ* from cells carrying a mutant copy and enables their relative quantification with next-generation sequencing; thus, even though the physical distribution of these mutations *in situ* remains unknown, we are effectively able to interrogate very large numbers of cells that could not be feasibly analyzed with single-cell sequencing based on current costs. Rahbari et al. (2016) suggested that many of these low-level mosaic mutations arose spontaneously in the parents during embryogenesis (particularly during expansion of primordial germ cell precursors). Although individual families in their study clearly exhibited different propensities to pass on such mosaic mutations, the underlying cause for the variation remains enigmatic. We propose that one contributor to variation in mutation burden among individuals is differential exposure to environmental mutagens during development.

The doses used in this study are higher than the levels of BaP to which most individuals in the general population would be exposed. The International Agency for Research on Cancer (IARC) has concluded that dietary exposure to BaP may be as high as 17 μg/person/day, or 0.28 μg/kg/day for a 60-kg human (IARC 2010). This dose equates to 1.9 μg/kg/day in mice after an allometric interspecies correction (U.S. EPA 2011), or 300-fold less than the lowest dose inducing mutations in the brains of developing mice. It should be noted that humans are exposed to complex mixtures of multiple chemicals through both oral and inhalation routes. Occupational exposures or lifestyle factors may result in highly exposed individuals, and because the pharmacokinetics of these complex mixtures and their interactions with the placenta and fetus are not well known, we believe that further research in this area is warranted.

## Conclusion

In summary, this study provides clear evidence that *in utero* exposure to a common environmental pollutant induces somatic mosaicism in tissues originating from all three germ layers and germline mosaicism in the F1 generation. Thus, *in utero* development represents a sensitive window for the genesis of mosaicism via environmental exposures to chemical mutagens, and the potential health consequences apply not only to the developing organisms, but also to their progeny, which can ultimately influence the genetic health of an entire species.

## Supplemental Material

(263 KB) PDF

## References

[r1] Autrup H (1993). Transplacental transfer of genotoxins and transplacental carcinogenesis.. Environ Health Perspect.

[r2] Beal MA Gagné R Williams A Marchetti F Yauk CL 2015 Characterizing benzo[*a*]pyrene-induced *lacZ* mutation spectrum in transgenic mice using next-generation sequencing. BMC Genomics 16 812, doi:10.1186/s12864-015-2004-4 26481219 PMC4617527

[r3] Biesecker LG Spinner NB 2013 A genomic view of mosaicism and human disease. Nat Rev Genet 14 307 320, doi:10.1038/nrg3424 23594909

[r4] Buscariollo DL Fang X Greenwood V Xue H Rivkees SA Wendler CC 2014 Embryonic caffeine exposure acts via A1 adenosine receptors to alter adult cardiac function and DNA methylation in mice. PLoS One 9 e87547, doi:10.1371/journal.pone.0087547 24475304 PMC3903656

[r5] Campbell IM Shaw CA Stankiewicz P Lupski JR 2015 Somatic mosaicism: implications for disease and transmission genetics. Trends Genet 31 382 392, doi:10.1016/j.tig.2015.03.013 25910407 PMC4490042

[r6] Campbell IM Yuan B Robberecht C Pfundt R Szafranski P McEntagart ME 2014 Parental somatic mosaicism is underrecognized and influences recurrence risk of genomic disorders. Am J Hum Genet 95 173 182, doi:10.1016/j.ajhg.2014.07.003 25087610 PMC4129404

[r7] Chepelev NL Moffat ID Bowers WJ Yauk CL 2015 Neurotoxicity may be an overlooked consequence of benzo[*a*]pyrene exposure that is relevant to human health risk assessment. Mutat Res Rev Mutat Res 764 64 89, doi:10.1016/j.mrrev.2015.03.001 26041267

[r8] Cohen ASA Wilson SL Trinh J Ye XC 2014 Detecting somatic mosaicism: considerations and clinical implications. Clin Genet 87 554 562, doi:10.1111/cge.12502 25223253

[r9] De S 2011 Somatic mosaicism in healthy human tissues. Trends Genet 27 217 223, doi:10.1016/j.tig.2011.03.002 21496937

[r10] Edwards JL, Klein RE (1961). Cell renewal in adult mouse tissues.. Am J Pathol.

[r11] Erickson RP 2010 Somatic gene mutation and human disease other than cancer: an update. Mutat Res 705 96 106, doi:10.1016/j.mrrev.2010.04.002 20399892

[r12] Fowler PA Dorà NJ McFerran H Amezaga MR Miller DW Lea RG 2008 *In utero* exposure to low doses of environmental pollutants disrupts fetal ovarian development in sheep. Mol Hum Reprod 14 269 280, doi:10.1093/molehr/gan020 18436539 PMC2408934

[r13] Gartner A, Milstein S, Ahmed S, Hodgkin J, Hengartner MO (2000). A conserved checkpoint pathway mediates DNA damage–induced apoptosis and cell cycle arrest in *C. elegans*.. Mol Cell.

[r14] Gossen JA Molijn AC Douglas GR Vijg J 1992 Application of galactose-sensitive *E. coli* strains as selective hosts for LacZ^–^ plasmids. Nucleic Acids Res 20 3254, doi:10.1093/nar/20.12.3254 1620626 PMC312470

[r15] IARC (International Agency for Research on Cancer) (2010). Some non-heterocyclic polycyclic aromatic hydrocarbons and some related exposures.. IARC Monogr Eval Carcinog Risk Hum.

[r16] Keohavong P, Thilly WG (1992). Determination of point mutational spectra of benzo[*a*]pyrene-diol epoxide in human cells.. Environ Health Perspect.

[r17] Lambert IB Singer TM Boucher SE Douglas GR 2005 Detailed review of transgenic rodent mutation assays. Mutat Res 590 1 280, doi:10.1016/j.mrrev.2005.04.002 16081315

[r18] Lodato MA Woodworth MB Lee S Evrony GD Mehta BK Karger A 2015 Somatic mutation in single human neurons tracks developmental and transcriptional history. Science 350 94 98, doi:10.1126/science.aab1785 26430121 PMC4664477

[r19] Lu LJ, Disher RM, Reddy MV, Randerath K (1986). ^32^P-postlabeling assay in mice of transplacental DNA damage induced by the environmental carcinogens safrole, 4-aminobiphenyl, and benzo(a)pyrene.. Cancer Res.

[r20] Lupski JR 2013 Genetics. Genome mosaicism—one human, multiple genomes. Science 341 358 359, doi:10.1126/science.1239503 23888031

[r21] Lupski JR (2015). Structural variation mutagenesis of the human genome: impact on disease and evolution.. Environ Mol Mutagen.

[r22] MacKenzie KM, Angevine DM (1981). Infertility in mice exposed in utero to benzo(a)pyrene.. Biol Reprod.

[r23] McCallister MM Maguire M Ramesh A Aimin Q Liu S Khoshbouei H 2008 Prenatal exposure to benzo(*a*)pyrene impairs later-life cortical neuronal function. Neurotoxicology 29 846 854, doi:10.1016/j.neuro.2008.07.008 18761371 PMC2752856

[r24] Mitiku N, Baker JC (2007). Genomic analysis of gastrulation and organogenesis in the mouse.. Dev Cell.

[r25] Mocarelli P Gerthoux PM Needham LL Patterson DG Jr Limonta G Falbo R 2011 Perinatal exposure to low doses of dioxin can permanently impair human semen quality. Environ Health Perspect 119 713 718, doi:10.1289/ehp.1002134 21262597 PMC3094426

[r26] Modgil S Lahiri DK Sharma VL Anand A 2014 Role of early life exposure and environment on neurodegeneration: implications on brain disorders. Transl Neurodegener 3 9, doi:10.1186/2047-9158-3-9 24847438 PMC4028099

[r27] Moffat I Chepelev NL Labib S Bourdon-Lacombe J Kuo B Buick JK 2015 Comparison of toxicogenomics and traditional approaches to inform mode of action and points of departure in human health risk assessment of benzo[*a*]pyrene in drinking water. Crit Rev Toxicol 45 1 43, doi:10.3109/10408444.2014.973934 PMC4521608 25605026

[r28] O’Brien JM Beal MA Gingerich JD Soper L Douglas GR Yauk CL 2014 Transgenic rodent assay for quantifying male germ cell mutant frequency. J Vis Exp 90 e51576, doi:10.3791/51576 PMC4692354 25145276

[r29] O’Brien JM, Beal MA, Yauk CL, Marchetti F (2016a). Benzo(a)pyrene is mutagenic in mouse spermatogonial stem cells and dividing spermatogonia.. Toxicol Sci.

[r30] O’Brien JM Beal MA Yauk CL Marchetti F 2016b Next generation sequencing of benzo(a)pyrene-induced *lacZ* mutants identifies a germ cell-specific mutation spectrum. Sci Rep 6 36743, doi:10.1038/srep36743 27829668 PMC5103183

[r31] O’Brien JM Walker M Sivathayalan A Douglas GR Yauk CL Marchetti F 2015 Sublinear response in *lacZ* mutant frequency of Muta^TM^Mouse spermatogonial stem cells after low dose subchronic exposure to N-ethyl-N-nitrosourea. Environ Mol Mutagen 56 347 355, doi:10.1002/em.21932 25598316 PMC6680333

[r32] OECD (Organization for Economic Cooperation and Development) 2011 Test No. 488: Transgenic Rodent Somatic and Germ Cell Gene Mutation Assays. OECD Guidelines for the Testing of Chemicals, Section 4., doi: 10.1787/9789264203907-en

[r33] Poduri A Evrony GD Cai X Walsh CA 2013 Somatic mutation, genomic variation, and neurological disease. Science 341 1237758, doi:10.1126/science.1237758 23828942 PMC3909954

[r34] Rahbari R Wuster A Lindsay SJ Hardwick RJ Alexandrov LB Al Turki S 2016 Timing, rates and spectra of human germline mutation. Nat Genet 48 126 133, doi:10.1038/ng.3469 26656846 PMC4731925

[r35] Ritz C Ruminski W Hougaard KS Wallin H Vogel U Yauk CL 2011 Germline mutation rates in mice following *in utero* exposure to diesel exhaust particles by maternal inhalation. Mutat Res 712 55 58, doi:10.1016/j.mrfmmm.2011.04.007 21570989

[r36] Rübe CE Zhang S Miebach N Fricke A Rübe C 2011 Protecting the heritable genome: DNA damage response mechanisms in spermatogonial stem cells. DNA Repair (Amst) 10 159 168, doi:10.1016/j.dnarep.2010.10.007 21123119

[r37] Russell LB (1999). Significance of the perigametic interval as a major source of spontaneous mutations that result in mosaics.. Environ Mol Mutagen.

[r38] Russell LB, Bangham JW, Stelzner KF, Hunsicker PR (1988). High frequency of mosaic mutants produced by *N*-ethyl-*N*-nitrosourea exposure of mouse zygotes.. Proc Natl Acad Sci U S A.

[r39] Russell LB, Russell WL (1992). Frequency and nature of specific-locus mutations induced in female mice by radiations and chemicals: a review.. Mutat Res.

[r40] Sanders SJ Murtha MT Gupta AR Murdoch JD Raubeson MJ Willsey AJ 2012 *De novo* mutations revealed by whole-exome sequencing are strongly associated with autism. Nature 485 237 241, doi:10.1038/nature10945 22495306 PMC3667984

[r41] Spinner NB Conlin LK 2014 Mosaicism and clinical genetics. Am J Med Genet C Semin Med Genet 166C 397 405, doi:10.1002/ajmg.c.31421 25424979

[r42] Tomasetti C Vogelstein B 2015 Cancer etiology. Variation in cancer risk among tissues can be explained by the number of stem cell divisions. Science 347 78 81, doi:10.1126/science.1260825 25554788 PMC4446723

[r43] U.S. EPA (U.S. Environmental Protection Agency) (2011). Recommended Use of Body Weight^3/4^ as the Default Method in Derivation of the Oral Reference Dose. EPA/100/R11/0001.. https://www.epa.gov/sites/production/files/2013-09/documents/recommended-use-of-bw34.pdf.

[r44] Vogelstein B Papadopoulos N Velculescu VE Zhou S Diaz LA Jr Kinzler KW 2013 Cancer genome landscapes. Science 339 1546 1558, doi:10.1126/science.1235122 23539594 PMC3749880

[r45] Wu S Powers S Zhu W Hannun YA 2016 Substantial contribution of extrinsic risk factors to cancer development. Nature 529 43 47, doi:10.1038/nature16166 26675728 PMC4836858

[r46] Yuen RKC Thiruvahindrapuram B Merico D Walker S Tammimies K Hoang N 2015 Whole-genome sequencing of quartet families with autism spectrum disorder. Nat Med 21 185 191, doi:10.1038/nm.3792 25621899

