# Supplementary material for: In Utero Exposure to Benzo[a]Pyrene Increases Mutation Burden in the Soma and Sperm of Adult Mice
Source: Environ Health Perspect. 2016 Jul 22;125(1):82–8. doi: 10.1289/EHP211 (PMC5226697; doi:10.1289/EHP211)
Supplement: (263 KB) PDF [file EHP211.s001.acco.pdf]

Environ Health Perspect
DOI: 10.1289/EHP211

Note to readers with disabilities: *EHP* strives to ensure that all journal content is accessible to all readers. However, some figures and Supplemental Material published in *EHP* articles may not conform to 508 standards due to the complexity of the information being presented. If you need assistance accessing journal content, please contact ehp508@niehs.nih.gov. Our staff will work with you to assess and meet your accessibility needs within 3 working days.

Supplemental Material

In Utero Exposure to Benzo[a]Pyrene Increases Mutation Burden in the Soma and Sperm of Adult Mice

Matthew J. Meier, Jason M. O'Brien, Marc A. Beal, Beverly Allan, Carole L. Yauk, and Francesco Marchetti

Table of Contents

Figure S1. Number of mutations occurring after various animal exposures to BaP normalized to total quantity of BaP exposure (see Table S2). Each data point represents the mean value for a different dose group. BM: bone marrow; LV: liver; BR: brain.

Figure S2. Dose-response modeling of the brain of F1 males exposed *in utero*. The BMDS software v2.6 was used with a BMR of 1 standard deviation with non-constant variance that adequately modeled the data. These parameters were chosen to allow comparison with existing cancer benchmark dose analysis from (Moffat et al. 2015).

Table S1. Summary of *lacZ* mutant frequency in somatic and germ tissues of Muta™Mouse males exposed to BaP *in utero* during the period of organogenesis.

Table S2. Mutant frequencies expressed per cumulative dose of BaP administered in this study and in previously exposed adult Muta™Mouse males. The source of each *lacZ* mutant frequency data is stated below.

Table S3. Comparison of clonal expansion of *lacZ* mutants in control mice and mice exposed to BaP either *in utero* or as adults.

Table S4. Non-normalized raw data output from computer-assisted sperm analysis (CASA).

Table S5. Raw data used to generate Figure 2A and Figure 2D, liver and testes somatic indices.

Supporting References

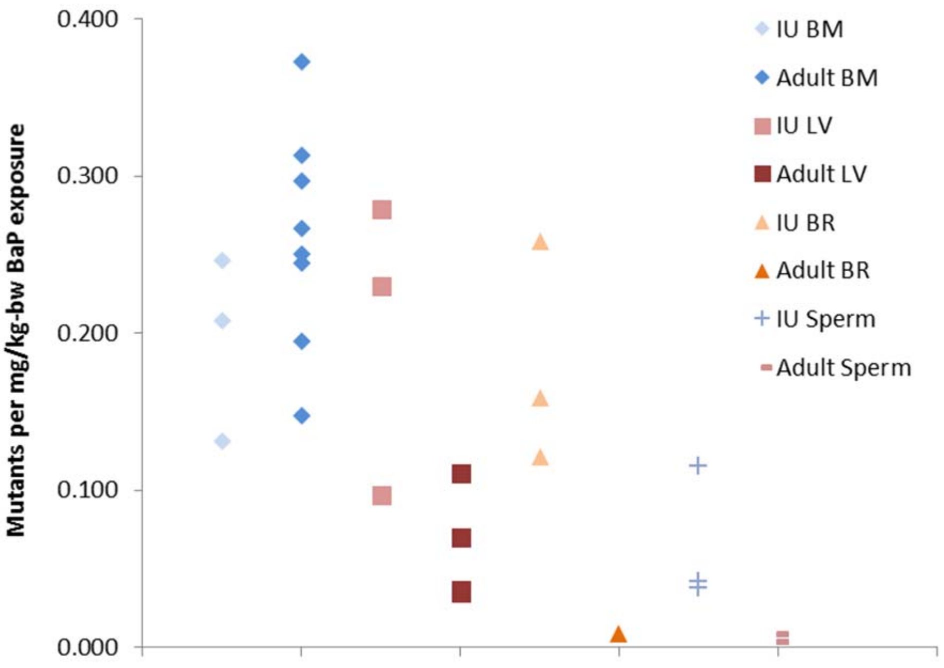

Figure S1. Number of mutations occurring after various animal exposures to BaP normalized to total quantity of BaP exposure (see Table S2). Each data point represents the mean value for a different dose group. BM: bone marrow; LV: liver; BR: brain.

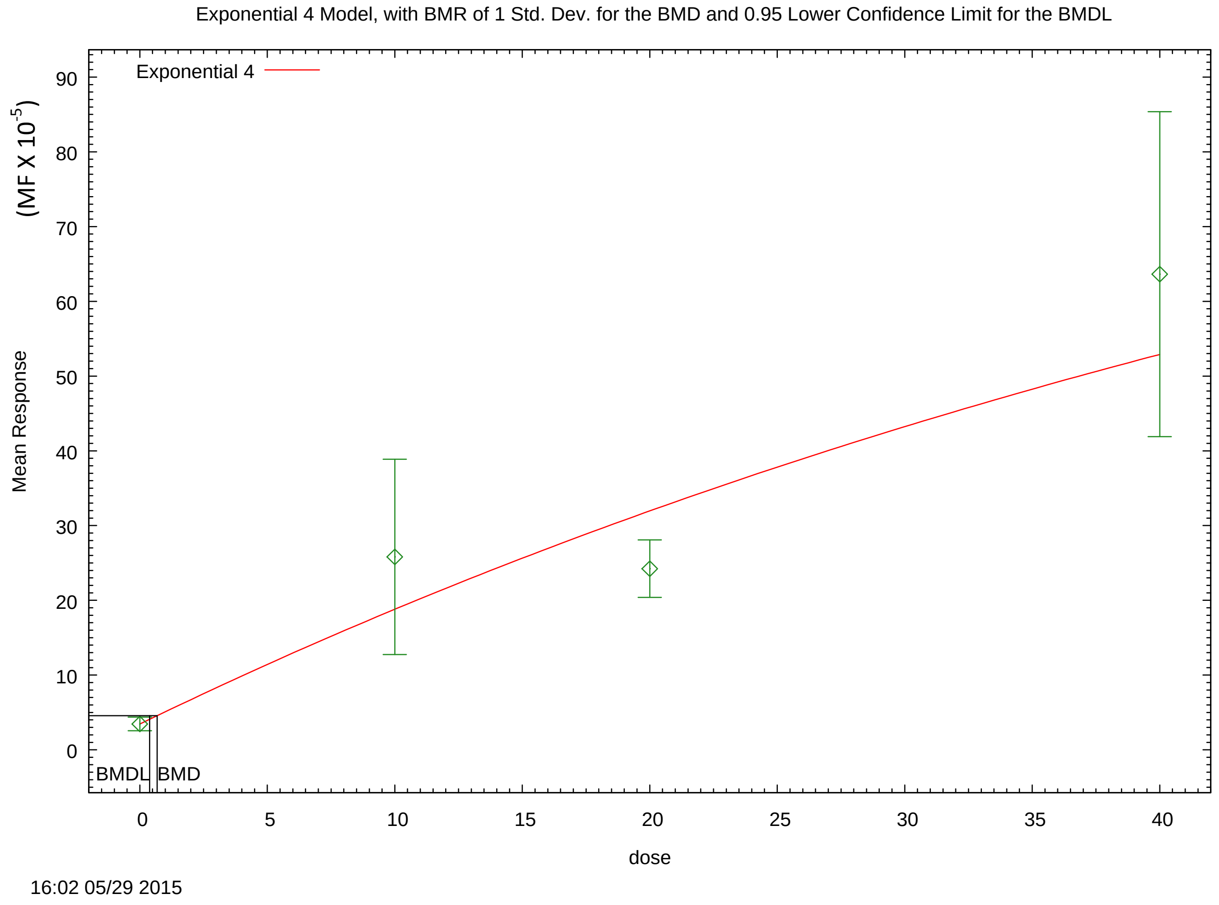

Figure S2. Dose-response modeling of the brain of F1 males exposed *in utero*. The BMDS software v2.6 was used with a BMR of 1 standard deviation with non-constant variance that adequately modeled the data. These parameters were chosen to allow comparison with existing cancer benchmark dose analysis from (Moffat et al. 2015).

Table S1. Summary of *lacZ* mutant frequency in somatic and germ tissues of Muta™Mouse males exposed to BaP *in utero* during the period of organogenesis.

Tissue	Dose administered to dam (mg/kg/d)	No of F1 animals	Avg[a] MF (×10^5)	SE[b]	P-value[c]	Total Mutants	Total Plaque Forming Units
**Bone marrow**	0	16	6.2	2.19	–	169	3,148,992
	10	9	13.1	4.03	0.1323	379	2,561,041
	20	18	41.6	5.46	<0.0001	1,874	4,205,448
	40	8	98.5	14.05	<0.0001	1,172	1,291,372
**Brain**	0	8	3.5	0.87	–	67	2,037,037
	10	5	25.8	3.13	<0.0001	348	1,290,543
	20	15	24.2	2.24	<0.0001	582	2,330,433
	40	8	63.6	4.86	<0.0001	659	1,146,192
**Liver**	0	14	3.4	1.18	–	101	2,584,069
	10	11	9.7	1.91	0.0378	252	2,533,872
	20	15	45.9	3.83	<0.0001	1,347	2,920,372
	40	7	111.5	8.00	<0.0001	1,508	1,478,244
**Caudal sperm**	0	27	2.9	0.31	–	266	10,285,084
	10	17	3.8	0.43	0.1509	263	7,261,336
	20	11	8.5	0.97	<0.0001	254	3,188,918
	40	2	46.3	39.58	0.0003	7	12,922
**Whole testes**	0	6	2.1	0.92	–	40	1,845,468
	10	4	7.2	1.77	0.0888	110	1,594,804
	20	12	21.8	2.02	<0.0001	770	3,681,859
	40	3	77.6	8.12	<0.0001	680	862,129

[a]Arithmetic mean of mutant frequency (MF) of all animals in group

[b]Standard error (SE) calculated using the esticon function in the doBy R package

[c]Generalized linear model in R with quasi-Poisson distribution and Bonferonni correction

Table S2. Mutant frequencies expressed per cumulative dose of BaP administered in this study and in previously exposed adult Muta™Mouse males. The source of each *lacZ* mutant frequency data is stated below.

**Tissue**	**Exposure**	**Length of exposure + time until euthanasia**	**Avg. mutants per mg/kg-bw**	**Fold difference between *in utero* and adult BaP exposure**
Bone marrow	*In utero*	10 d + 10 wk	0.195	**0.75**
Brain	*In utero*	10 d + 10 wk	0.179	**19.3**
Liver	*In utero*	10 d + 10 wk	0.202	**3.2**
Sperm	*In utero*	10 d + 10 wk	0.066	**11.4**
Bone marrow	Adult[a,b,c]	28 d + 3 d	0.261	
Brain	Adult[c]	5 d + 14 d	0.009	
Liver	Adult[d]	28 d + 3 d	0.063	
Sperm	Adult[a]	28 d + 42 d	0.006	

[a]O'Brien et al. (2016)

[b]Beal et al. (2015).

[c]Hakura et al. (1998).

[d]Lemieux et al. (2011).

Table S3. Comparison of clonal expansion of *lacZ* mutants in control mice and mice exposed to BaP either *in utero* or as adults.

**Tissue (study)**	**Exposure**	**# unique mutations**	**# clonally expanded**	**% clonally expanded**	**Ratio (BaP:control)**	**Fisher's exact test p-value**
Bone Marrow (*in utero*)	Control	50	11	22%	1.55	0.1171
	Treated	155	53	34%		
Bone Marrow (adult)[a]	Control	152	11	8%	1.25	0.5362
	Treated	854	78	10%		
Brain (*in utero*)	Control	49	2	4%	4.27	**0.0153**
	Treated	235	41	17%		
Caudal sperm (*in utero*)	Control	54	8	15%	1.97	**0.0792**
	Treated	65	19	29%		
Caudal sperm (adult)[b]	Control	131	18	16%	0.63	0.1247
	Treated	295	26	10%		
Liver (*in utero*)	Control	80	3	4%	2.07	0.2901
	Treated	193	15	8%		

[a]Beal et al. (2015).

[b]O'Brien et al. (2016)

Table S4. Non-normalized raw data output from computer-assisted sperm analysis (CASA).

ID	Study	Dose Group	TOTAL COUNT	TOTAL CONC	MOTILE COUNT	MOTILE %	PROGRESSIVE %	VAP	VSL	VCL	ALH	BCF	STR	LIN	ELONGATION	SIZE
1-1m	BaP IU-1	Control	2937.5	15.0	1588.5	54.5	21.5	118.9	87.8	242.1	12.1	41.5	68.5	35.0	51.5	37.6
3-1m	BaP IU-1	Control	3236.0	11.2	1514.5	48.0	17.5	112.2	81.8	226.2	12.0	38.6	67.5	34.5	56.5	30.4
5-1m	BaP IU-1	Control	2312.0	11.8	1055.0	45.5	17.0	107.5	78.9	210.4	11.6	38.6	66.0	35.0	58.0	29.9
5-2m	BaP IU-1	Control	2780.5	7.1	603.0	21.5	7.5	77.4	58.0	152.8	9.0	35.8	71.5	38.0	62.5	25.4
7-1m	BaP IU-1	Control	3996.0	10.2	1849.5	46.0	16.0	102.5	74.8	205.3	11.4	38.8	65.5	34.5	58.0	26.8
7-3m	BaP IU-1	Control	2419.5	12.4	1259.0	52.0	22.0	124.6	93.6	247.0	12.0	40.4	70.0	37.0	55.5	32.7
7-4m	BaP IU-1	Control	2469.5	7.6	871.0	40.0	18.0	110.8	82.8	230.9	11.2	41.4	69.5	35.5	52.0	31.3
7-5m	BaP IU-1	Control	3287.5	11.8	1473.5	46.0	17.5	107.9	79.5	217.1	11.3	39.7	67.5	35.5	57.0	30.2
9-1m	BaP IU-1	Control	4633.0	16.2	1980.5	44.5	14.5	109.3	78.7	218.4	12.9	38.0	66.0	34.5	62.0	26.6
9-2m	BaP IU-1	Control	3848.0	13.0	1800.5	48.0	17.0	104.1	74.0	209.1	11.6	39.0	63.0	32.5	54.5	32.2
11-1m	BaP IU-1	Control	2717.0	9.5	1271.0	47.0	15.5	92.9	64.8	202.4	10.7	40.6	63.0	30.5	54.0	28.8
11-2m	BaP IU-1	Control	2753.5	11.3	1220.0	44.0	16.5	98.2	72.9	191.8	10.6	39.3	66.0	35.0	53.5	29.6
11-3m	BaP IU-1	Control	3490.5	7.1	1504.5	43.0	14.0	88.1	62.7	183.3	10.7	39.3	64.0	33.0	56.0	26.7
23CM1	BaP IU-2	Control	1865.0	3.8	898.0	46.0	32.5	118.1	81.5	271.3	14.2	38.0	65.5	30.5	61.5	22.1
26CM1	BaP IU-2	Control	1510.0	4.1	632.5	41.0	21.5	84.7	46.0	169.0	10.5	29.8	55.0	28.5	54.5	32.5
27CM1	BaP IU-2	Control	2475.5	6.7	1759.0	71.0	47.0	125.9	86.2	273.4	13.7	40.3	65.5	32.0	47.5	46.6
29CM1	BaP IU-2	Control	2350.5	6.4	1368.5	58.0	40.0	122.2	87.7	266.3	13.6	40.3	67.5	32.0	45.0	44.4
30CM1	BaP IU-2	Control	2307.0	6.3	1542.5	66.5	47.5	118.6	87.6	253.3	12.4	42.5	69.0	33.5	45.0	45.7
4-1m	BaP IU-1	BaP 10	2187.0	5.6	928.5	42.0	18.0	109.1	84.8	205.8	10.9	38.1	71.0	39.0	57.0	25.8
4-3m	BaP IU-1	BaP 10	2531.5	6.5	1248.0	48.5	18.0	97.5	72.8	198.5	11.0	36.7	70.5	37.5	56.0	25.5
6-1m	BaP IU-1	BaP 10	3013.0	10.7	1411.0	46.0	16.0	123.2	89.0	246.9	13.4	38.0	65.5	34.0	64.5	22.4
6-2m	BaP IU-1	BaP 10	2715.0	13.8	1275.0	46.5	18.0	122.2	90.6	237.2	12.2	39.8	67.0	35.5	57.5	30.9
6-4m	BaP IU-1	BaP 10	3394.5	8.7	1700.0	49.5	16.0	105.5	74.7	220.0	11.9	39.7	64.5	32.0	56.5	27.6
8-1m	BaP IU-1	BaP 10	2584.0	13.2	1084.5	42.0	16.0	112.9	83.8	214.1	12.1	37.7	66.0	35.5	61.0	25.8
10-1m	BaP IU-1	BaP 10	2819.0	11.5	1210.0	43.0	17.5	100.1	75.1	197.4	10.6	39.6	66.5	35.0	54.5	30.6
10-2m	BaP IU-1	BaP 10	4299.5	8.8	1659.0	38.0	12.0	90.3	62.4	190.5	11.1	38.3	62.0	31.5	59.5	24.3
12-1m	BaP IU-1	BaP 10	3140.5	8.0	1590.5	50.5	19.5	103.9	76.7	211.8	11.5	39.6	68.5	34.5	54.0	29.6

28BM1	BaP IU-2	BaP 20	408.5	1.2	38.5	9.5	6.5	68.9	54.3	125.2	7.8	31.1	74.0	44.5	45.5	31.2
29BM1	BaP IU-2	BaP 20	1338.0	3.6	324.5	24.5	17.0	116.3	88.9	218.0	9.9	38.9	72.0	41.0	47.0	35.3
29BM2	BaP IU-2	BaP 20	708.0	2.0	137.5	19.5	14.0	118.5	89.4	234.0	11.9	30.7	70.5	38.0	46.0	31.0
29BM3	BaP IU-2	BaP 20	1553.0	4.2	827.0	53.5	39.0	122.5	96.9	251.3	11.2	42.5	74.0	38.0	44.0	44.5
29BM4	BaP IU-2	BaP 20	286.0	0.8	21.5	8.0	6.5	67.2	55.6	123.4	6.9	32.3	79.0	46.0	51.5	29.0
29BM5	BaP IU-2	BaP 20	1080.5	3.0	633.5	58.0	46.0	128.6	103.1	256.8	10.8	43.0	75.5	39.5	39.0	40.5
30BM1	BaP IU-2	BaP 20	2683.0	7.3	1084.0	40.5	25.0	100.0	73.2	212.1	12.1	38.1	68.5	33.5	53.5	32.4
33BM1	BaP IU-3	BaP 20	1026.5	4.2	103.0	10.0	6.0	87.9	61.0	188.1	9.0	35.2	64.0	34.5	53.5	26.9
33BM2	BaP IU-3	BaP 20	1497.0	6.2	206.0	14.0	10.0	93.5	71.6	185.9	8.8	38.0	71.0	39.0	45.5	37.7
34BM1	BaP IU-3	BaP 20	1103.7	4.5	9.3	3.7	1.0	16.2	9.1	32.2	1.2	17.3	39.0	24.0	51.7	37.2
34BM2	BaP IU-3	BaP 20	289.0	1.2	4.0	1.0	0.0	37.2	21.0	97.5	0.0	26.0	64.0	24.0	81.0	3.5
34BM3	BaP IU-3	BaP 20	188.0	0.8	7.0	4.0	1.0	25.9	15.3	62.9	10.5	27.7	64.0	31.0	91.0	6.0
34BM4	BaP IU-3	BaP 20	286.0	1.2	8.0	2.5	2.0	65.6	54.1	111.3	6.8	32.3	76.5	46.5	51.5	33.1
35BM1	BaP IU-3	BaP 20	1363.0	5.6	415.5	30.5	19.5	112.3	84.2	229.3	11.2	42.0	68.5	36.5	51.0	29.4
35BM2	BaP IU-3	BaP 20	817.0	3.4	127.5	15.5	10.5	76.8	53.7	149.6	7.8	33.3	68.0	38.5	51.0	31.7
36BM1	BaP IU-3	BaP 20	1386.5	5.7	445.5	32.0	22.0	132.6	100.7	257.0	12.4	38.7	71.5	38.5	46.5	32.8
36BM2	BaP IU-3	BaP 20	322.0	1.8	27.0	7.0	4.5	117.8	94.5	201.1	7.3	40.1	70.5	49.0	52.5	27.1
25AM1	BaP IU-2	BaP 40	258.5	0.7	15.0	6.0	4.0	66.6	46.9	123.4	6.2	31.5	68.5	39.0	58.0	20.6
27AM1	BaP IU-2	BaP 40	150.0	0.4	2.0	1.0	0.0	18.0	13.6	30.5	0.0	45.0	76.0	45.0	78.0	9.2
27AM2	BaP IU-2	BaP 40	257.0	0.7	9.0	3.0	0.0	11.6	7.8	36.5	0.0	20.0	39.0	19.0	41.0	3.8
30AM1	BaP IU-2	BaP 40	228.0	0.6	10.0	4.0	2.0	47.6	30.7	114.6	8.6	42.1	65.5	29.0	60.0	20.9
32AM1	BaP IU-3	BaP 40	66.0	0.4	0.5	0.5	0.0	0.0	0.0	0.0	0.0	0.0	0.0	0.0	0.0	0.0
32AM2	BaP IU-3	BaP 40	164.5	0.8	0.0	0.0	0.0	0.0	0.0	0.0	0.0	0.0	0.0	0.0	0.0	0.0
32AM3	BaP IU-3	BaP 40	179.0	0.7	3.0	2.0	0.0	11.2	10.1	11.9	0.0	13.5	45.0	42.0	33.0	6.9
36AM1	BaP IU-3	BaP 40	141.0	0.6	0.5	0.5	0.5	20.5	14.6	44.0	1.6	0.0	35.5	16.5	11.0	8.8

Table S5. Raw data used to generate Figure 2A and Figure 2D, liver and testes somatic indices.

Dose Group	Animal weight (g)	Liver weight (g)	Left testis weight (g)	Right testis weight (g)
Control	27.2	0.977	0.105	0.144
Control	26.1	1.189	0.100	0.108
Control	21.8	1.295	0.118	0.116
Control	32.3	1.233	0.066	0.064
Control	26.7	1.163	0.106	0.107
Control	28.3	0.989	0.097	0.097
Control	29.4	0.945	0.100	0.099
Control	30.0	1.125	0.121	0.199
Control	27.1	1.044	0.109	0.114
Control	33.3	1.093	0.118	0.135
Control	23.7	0.985	0.095	0.099
Control	25.1	1.225	0.111	0.114
Control	24.0	0.926	0.099	0.092
Control	33.0	1.092	0.100	0.108
Control	23.9	0.901	0.094	0.100
Control	24.6	1.028	0.107	0.033
Control	50.8	1.183	0.124	0.132
Control	32.8	1.388	0.127	0.125
BaP 10	23.6	1.094	0.069	0.071
BaP 10	23.9	1.042	0.013	0.032
BaP 10	24.0	0.908	0.167	0.172
BaP 10	28.1	0.970	0.099	0.093
BaP 10	25.1	1.120	0.113	0.112
BaP 10	25.9	1.064	0.097	0.105
BaP 10	24.0	1.253	0.091	0.094
BaP 10	25.2	0.991	0.086	0.092
BaP 10	28.5	1.202	0.107	0.104
BaP 10	30.5	1.408	0.112	0.089
BaP 10	23.2	0.894	0.071	0.076

BaP 20	32.1	1.201	0.046	0.042
BaP 20	35.4	1.355	0.073	0.072
BaP 20	28.7	1.165	0.068	0.079
BaP 20	30.5	1.121	0.025	0.038
BaP 20	23.6	0.834	0.050	0.059
BaP 20	32.7	1.251	0.087	0.088
BaP 20	28.6	2.540	0.039	0.043
BaP 20	25.0	2.648	0.049	0.050
BaP 20	23.8	2.530	0.036	0.043
BaP 20	23.9	0.874	0.035	0.043
BaP 20	20.7	0.828	0.042	0.030
BaP 20	24.3	0.964	0.026	0.033
BaP 20	32.0	1.237	0.059	0.055
BaP 20	27.2	2.514	0.046	0.054
BaP 20	27.7	1.089	0.061	0.052
BaP 20	25.1	0.966	0.044	0.056
BaP 40	23.8	0.954	0.017	0.017
BaP 40	25.4	1.050	0.023	0.024
BaP 40	23.4	0.935	0.015	0.013
BaP 40	22.6	0.795	0.016	0.022
BaP 40	27.0	1.046	0.028	0.026
BaP 40	20.5	0.805	0.018	0.015
BaP 40	28.8	1.243	0.031	0.031
BaP 40	31.3	1.152	0.022	0.018
BaP 40	25.2	0.943	0.026	0.039

Supporting References

Beal MA, Gagné R, Williams A, Marchetti F, Yauk CL. 2015. Characterizing Benzo[a]pyrene-induced lacZ mutation spectrum in transgenic mice using next-generation sequencing. BMC Genomics 16:812; doi:10.1186/s12864-015-2004-4.

Hakura A, Tsutsui Y, Sonoda J, Kai J, Imade T, Shimada M, et al. 1998. Comparison between in vivo mutagenicity and carcinogenicity in multiple organs by benzo[a]pyrene in the lacZ transgenic mouse (Muta Mouse). Mutat. Res. 398: 123–30.

Lemieux CL, Douglas GR, Gingerich J, Phonethepswath S, Torous DK, Dertinger SD, et al. 2011. Simultaneous measurement of benzo[a]pyrene-induced Pig-a and lacZ mutations, micronuclei and DNA adducts in Muta™ Mouse. Environ. Mol. Mutagen. 52:756–65; doi:10.1002/em.20688.

Moffat I, Chepelev NL, Labib S, Bourdon-Lacombe J, Kuo B, Buick JK, et al. 2015. Comparison of toxicogenomics and traditional approaches to inform mode of action and points of departure in human health risk assessment of benzo[a]pyrene in drinking water. Crit. Rev. Toxicol. 45:1–43; doi:10.3109/10408444.2014.973934.

O'Brien JM, Beal MA, Yauk CL, Marchetti F. 2016. Benzo (a) pyrene is mutagenic in mouse spermatogonial stem cells and dividing spermatogonia. Toxicological Sciences Epub: May 13, 2016.
